# 

**Published:** 1848-12

**Authors:** 


### INDEX.

Absence of the globes of both eyes, case of 263
Abscess of the thoracic walls, Dr. Crisp's case of 560
Action of hydrocyanic acid on iodine 77
Account of a case of prolapse of the gravid uteris during labour, by E. F. Watson, M. D. 240
Academy of Medicine of Paris 590
Acidity and alkalinity of certain of of the human fluids in the state of health and disease, M. Andral on 706
American Medical Association 262, 361
" " " transactions of, bib. notice of 740
Amputation of diseased joints, influence of age on the results of 399
Anus, imperforate 69
Anasarca with albuminous urine, Dr. Herapath's case of, in an infant ten weeks old 101
Annual circulars and catalogues 122
Animal temperature 325
Anniversary discourse before the New York Academy of Medicine, by J. W. Francis, M. D., bib. notice of 357
Andral on general pathology 374
Annual address to the Chester county Medical Society, by W. Worthington, M. D., bib. notice of 437
Animal charcoal as an antidote, Dr. Rand on 528
Angina folliculosa of the pharanx, by M. Chomel 550
Annalist 359, 646
Analytical compendium, Neill's and Smith's, bib. notice of 683
Anatomico-physiological notes on the structure of the heart of the sturgeon and of the ray, by M. Parchappe, M. D. 729
Appointment of delegates to National and State Convention 200
Appointment, Medical in Philada. 306
Asclepias tuberosa butterfly weed, &c., by T. L. Lockwood, M. D. 367
Asiatic cholera, 199
" " treatment of 319, 695
Atropia, on the use of nitrate of, for producing dilatation of the pupils 652
Bank's case of extensive laceration of the liver and diaphragm from the back of a horse 534
Bartlett's inquiry into the degree of certainty in medicine, bib. notice of 686
Bennett on the treatment of phthisis pulmonalis by cod-liver oil 379
Bertolet's case of twins of extraordinary size 471
Bell and Stokes lectures on the theory and practice of physic, bib. notice of 480
Bibliographical Notices 20, 108, 190, 244, 296, 346, 423, 475, 535, 625, 679, 733
Blennorraghia, Record's lectures on 59
Blood on clothes, method of recognizing the presence of 397
Blisters, note on the dressing of 695
Boussingault's researches on the influence of salt added to the food in the development of animals 324
Bower's memoranda of anatomy, surgery, and physiology, bib. notice of 437
Bostwick's treatise on the nature and treatment of seminal diseases, impotency, &c., bib. notice of 679

Bostwick on the nature and treatment of venereal diseases, bib. notice of 679
British Parliament—evidence taken before the Select Committee on Medical Legislation 134
Bronchitis and pneumonia of infants, Golding's general remarks on 115
Brownbilli's case of spontaneous rupture of the uterus before labour 207
Brain, the human, Solly on, bib. notice of 248
Bryan on the nitrate of silver in croup 342
Bryan on the medical treatment of cataract 401
Burns, treatment of 78
Burrows on the disorders of the cerebral circulation, and on the connection between affections of the brain and diseases of the heart, bib. notice of 248

Cases occurring at the clinic of Jefferson Medical College. Reported by James Patterson, M. D. 13
Case of congenital absence of the globes of both eyes 263
Canstatt's handbuch der medicinischen klinik, bib. notice of 31
Catalogue of the medicinal plants, indigenous and exotic, growing in the State of New York, with a brief account of their composition, by C. Lee, M. D., bib. notice of 118
Catalogues and annual circulars 122
Calisaya extract, C. Ellis on 130
Case of a woman who, by means of galvano-puncture, recovered her speech after having lost it for 23 years, by M. Camino, M. D. 326
Case of a large tumour involving the superior maxillary bone, removed by Prof. Pancoast 472
Case of separation of the stomach from the œsophagus, by T. M. Flint 715
Cataract, medical treatment of, Dr. Bryan's remarks on 401
Caries of the parietes of the tympanum, producing menengitis, without destruction of the petrous portion of the temporal bone 445
Caries of the parietes of the tympanum, producing meningitis or cerebritis, in consequence of destruction of the osseous septum between its cavity and that of the cranium 446
Caries of the parietes of the tympanum, inducing phlebitis of the lateral sinus and internal jugular vein 448
Cancer of the stomach, with tubercular peritonitis, and dropsy of the peritoneum, necroscopy, and remarks, by Lawrence Turnbull, M. D. 724
Cholera 37
" and influenza 119
" Asiatic 199, 210, 438, 486, 645, 696
" Menneret's history of the, observed in Constantinople during the winter of 1847–8 320
" subsidence of, in Europe and Asia 328
" naphtha a cure for 518
" key to, by G. W. Maxwell 581
" in England 690
" in Russia 708
" progress of 761
" on an electrical phenomena observed in, by Dr. Atkinson 764
" and the health of London 766
Chloroform, remarks on, by L. Turnbull, M. D. 83
" in dentistry, by Dr. Buchanan 135
" Prof. Simpson on 145
" " " formula for 147
" Dr. Meigs' reply to Prof. Simpson's letter on 148
" in surgery and midwifery, by C. H. Cragin, M.D. 223
" death from the inhalation of 267
" Harrington's remarks on 295
" in chorea 328
" a test of simulated disease 329
" and other anæsthetic agents, local effects of 520
Chlorine, Dr. Coxe's reminiscences of the effects of, with the favourable result of copious bleeding 211
Chapman, professor 261
Children, lithotrity in 270
Churchill on the theory and practice of midwifery, bib. notice of 300
Christison's dispensatory, or commentaries on the pharmacopœias of Great Britain and the United States, bib. notice of 540
Chomel on angina folliculosa of the pharynx 550
Channing's treatise on etherization in childbirth, bib. notice of 737
Clinical instruction in Italian schools, 67
Clark's cases of the use of ether in obstetrical practice 153

Clary on erysipelas of the head and face 416
Constantinople, medical education at 77
Coates' case of discharge of a tooth from the ear 77
Concours for the chair of clinical surgery in the Academy of Medicine, vacant by the death of Prof. A. Bérard 133
Conventions, national and state, appointment of delegates to 200
Compound dislocation of the knee-joint, Dr. Costill's case of 291
Convention, state medical 306
Cod-liver oil, Dr. Bennett on the treatment of phthisis pulmonalis, by 379
Cod-liver oil, test for 710
Correction, 363
Cooper, Mr. Bransby on erysipelas 380
Costill's practical treatise on poisons, their symptoms, antidotes and mode of treatment, bib. notice of 485
Cold, Dr. Arnott on, as a means of producing local insensibility 566
Colleges, medical 646
Collodium or collodion 711
Crayons of nitrate of siver and potassa, 209
Creosote, use of, in mercurial salivation, Dr. Faulcon on 656
Cyst in the uterus of a child three months old, with another cyst like a corpus luteum in one of the ovaries 69
Cyclopedia of anatomy and physiology, edited by R. Todd, M. D., bib. notice of, 248

Datura stramonium as an emmenagogue, by B. F. Jones, M. D. 336
Death instantly following a blow on the mouth; by Hayes Kyd, Esq., &c. 61
Death from effusion of blood into the left lateral ventricle; report of the late illness, death and autopsy of the Rev. A. T. Tompkins, by Dr. Hamilton 129
Death from the inhalation of chloroform 267
Death, apparent—premature interment 458
Death, singular cause of 526
" accelerated by salivation in the third stage of tubercular consumption 713
Dentistry, chloroform in, by Dr. Buchanan 135
Descriptive catalogue of the anatomical museum of the Boston Society for medical improvement, by J. B. S. Jackson, M. D., bib. notice of 360
Deafness, a new mode of treating, by Mr. Yearsley 576
Deaf ness and diseases of the ear, nature and treatment of, and treatment of the deaf and dumb, by W. Dufton, M. D., bib. notice of 759
Discovery of a new anæsthetic agent more efficient than sulphuric ether, by J. Y. Simpson, M. D. 75
Discharge of a tooth from the ear, by M. Coates, M. D. 77
Dieffenbach and Liston 138
Disorders of the cerebral circulation, and connection between affections of the brain and diseases of the heart, by G. Burrows, M. D., bib. notice of 248
Diseases of children, Meigs' practical treatise on, bib. notice of 538
Dowler's researches on meteorology, bib. notice of 297
Donovan's observations on the peculiar diseases to which the famine of last year gave origin 381
Double birth, extraordinary, Dr. Askham's report of 700
Dunglison's practice of medicine, bib. notice of 33
Dunglison's dictionary of medical science, bib. notice of 639
Dunott's remarkable case of strangulated hernia, successfully treated 292
Dufton on the nature and treatment of deafness and diseases of the car, and treatment of the deaf and dumb, bib. notice of 759
Dyspepsia, Dr. Livezey on various forms of 279

Earle's history, description and statistics of the Bloomingdale Asylum for the insane, bib. notice of 432
Eclectic practitioners, or the so-called practical men 378
Editorial 34, 119, 198, 259, 308, 361, 438, 486, 542, 640, 690, 761
Editorial change 691
Œdema of the lower extremities in phthisis—extract of a letter from F. W. Lewis, M. D., to Prof. Mütter, dated Paris, Dec. 14, 1848 273
Ellis on calisaya extract 130
Elements of general pathology, by A. Stillé, M. D., bib. notice of 303

Electricity, heating power of low charges of 590
Electrical phenomena observed in cholera, by J. C. Atkinson, M. D. 764
Endocarditis 397
Ergot of rye, effects of, on the parturient female and her offspring 75
Ergot of rye a remedy for excessive dilatation of the pupil from belladonna 708
Erysipelas, Mr. Bransby Cooper on 383
" of the head and face, Dr. Clary on 416
Ether and the perchloride of formyle or chloroform, in surgical operations 18
Ether in obstetrical practice, Clark's cases of the use of 153
Etherization, Prof. Lindsley on 339
Etherization in childbirth, a treatise on, by Dr. W. Channing, bib, notice of 737
Expulsion of the entire ovum at the full time 68
Expulsion of two fœtuses of unequal size at the same time, with remarks on superfœtation, by E. Horlbeck, M. D. 125
Extra-uterine pregnancy, case of, by S. A. Peters 283
Extension of the lecture term by the medical colleges 262, 438, 487, 543

Fallopian tube, rupture of 272
Famine of the last year, observations on the peculiar diseases to which it gave origin 381
Faulcon on the use of creosote in mercurial salivation 656
Females and their diseases; a series of letters to his class, by Charles D. Meigs, M. D., bib. notice of 24
Fever, typhoid, as it prevailed in Germantown, Tennessee, during the fall of 1847, by R. L. Scruggs, M. D. 80
Fever, ship typhus, in New Orleans 266
" puerperal, by R. M. West, M. D. 315
Fever, puerperal, illustrations of the contagious nature of, &c. 506
Fear, effects of 460
Fisher on the nature and treatment of sea-sickness 54
Fistula in ano, treatment of 320
Flagg on neurology and sul. ether 15
Fluid, Ledoyen's disinfecting 34
Flint's case of separation of the stomach from the œsophagus 715
Francis' anniversary discourse before the New York Academy of Medicine, bib. notice of 357
Fracture of the orbit from a lead pencil 504
Fractures and wounds by fire-arms, Velpeau on the gravity and treatment of 557

Gall bladder, ossified 209
Galvanism and electro-magnetism, in the treatment of uterine hæmorrhage, prolapsus uteri, &c., by T. E. Waller, M. D. 237
Gardner's medical chemistry, for the use of students and the profession, bib. notice of 476
Generation, tracts on, translated from the German, by C. R. Gilman, M. D. 190
Gestation, interstitial extra-uterine 368
" utero, Dr. Clay's observations on 569
Gout in the common fowl 399
Gooch's account of the most important diseases peculiar to women, bib. notice of 634
Griffith on the blood and urine, bib. notice of 304
Graves, Dr., on a system of clinical medicine, bib. notice of 638
Gunshot wounds, Prof. Velpeau on 380
Gunshot wound, Dr. Eve's case of 549
Gun cotton, formula for the preparation of the ethereal solution of 649
Gutta percha, medicinal employment of 525
Gutta percha, or gum gettanea, by Solly and Soubeiran 552

Handbuch der medicinischen klinik, verfasst von Dr. C. Canstatt, ordentlichen, professor der medicin an der universität zu Erlangen und Mitgliede mehrerer gelehrter Gesellschaften, bib. notice of 31
Hamilton's observations on enlarged tonsils 42
Hamilton's case of death from effusion of blood into the left lateral ventricle 129
Hall on the theory of spasmo-paralysis in infants and adults, by M. Hall 331
Hastings' lectures on yellow fever, bib. notice of 346

Half-yearly abstract of the medical sciences, by W. H. Ranking, M.D., bib. nottce of 639
Hampden Sydney college 691
Hernia, remarkable case of, successfully treated, by Dr. Dunott 292
Hemorrhage, uterine, remarkable case of, terminating fatally, by F. L. Sewall, M. D. 341
Hereditary insanity 396
Heart, rupture of the 767
History, description and statistics of the Bloomingdale Asylum for the insane, by Pliny Earle, M. D., bib. notice of 432
Homœopathy and hydropathy outdone by the te-to-tum doctor of Arkansas 53
Horlbeck's case of the expulsion of two fœtuses of unequal size 125
Hospitals, United States Marine 166
Holmes' remarks on scurvy 364
" on malaria in connection with medical topography 493
Holmes on quinine 762
Hornet's nest 526
Hood's principles and practice of medicine, bib. notice of 635
Human anatomy, general and special, a system of, by E. Wilson, M. D., bib. notice of 640
Hydrocyanic acid, action of, on iodine 77
Hydrophobia, Smiley's case of 233
Hydrargyrum cum creta, and hydrargyrum cum magnesia of the Dublin pharmacopœia, Dr. Donovan on 702

Imperforate anus 69
Influenza and cholera 119
Influenza; 200
Introductory lectures 122, 262
" " a word on 396
Inoculation of medicines 324
Insanity not a result of the separate system of imprisonment 329
Insanity, hereditary 396
Interstitial extra-uterine gestation 368
Insane, on the minute attention to the comforts of the 399
Influence of age on the results of amputation for diseased joints 399
Intus-susception 523
Indian hemp, its attractive principle 524
Insoluble substances, absorption of 711
Incision of the urethra 766
Incarcerated hernia, reduction of 767
Iron, citrate of 649
Isopathy 397

Italian schools, clinical instruction in 67
Italy, medical congress of 456

Jarvis' practical physiology for the use of schools and families 197
Jackson's descriptive catalogue of the anatomical museum of the Boston Society for medical improvement bib. notice of 300
Jones on datura stramonium, as an emmenagogue 336

Key to cholera, by G. W. Maxwell 581

K[illegible]d's case of death, instantly following a blow in the mouth 64

Lallemand's practical treatise on the causes, symptoms and treatment of spermatorrhœa, bib. notice of 296
Lake on hydrogen in the electric fluid 371
Laceration, extensive, of the liver and diaphragm, from the kick of a horse, by Dr. E. C. Banks 534
Laughable 647
Lactation, prolonged, cases of 708
Ledoyen's disinfecting fluid 34
Lectures on the physical phenomena of living beings, by Carlo Matteucci, professor iu the University of Pisa, bib. notice of 110
Lee's catalogue of medicinal plants, indigenous and exotic, growing in the state of New York, with a brief account of their composition and medical qualities, bib. notice of 118
Lectures, introductory 262
" " a word on 396
" on general pathology, by Prof. Andral 374
Leeches, Soubeiran and Bouchardat on the method employed for disgorging 393
Lectures on the theory and practice of physic, by J. Bell, M. D., and W. Stokes, M. D., bib. notice of 480
Leibig's researches on the motion of the juices in the animal body, bib. notice of 483
Liston, successor to 122
" and Dieffenbach 138
Lithotrity in children 270
Livezy on various forms of dyspepsia 279
Lindsly on etherization 339
Literature, medical, in the United States, by a member of the National Medical Association 405
Lockwood on asclepia tuberosa, or butterfly weed, &c. 367

Materia medica and therapeutics, by Martyn Paine, M.D., &c., bib. notice of 20
Matteucci's lectures on the physical phenomena of living beings, bib. notice of 110
Malformation, anomalous case of, in a child-bearing woman 238
Mayne's dispensatory and therapeutical remembrancer, bib. notice of 301
Mammary abscesses, a point in the treatment of 460
Malaria in connection with medical topography, Dr. Holmes on 493
Malignant tumour of the os uteri, Dr. Arnott's case of 574
Malignant disease of the lungs and heart, Dr. Emmet's case of 588
Marriage, the philosophy of, by M. Ryan, M. D., bib. notice of 689
Materia medica, Bouchardat, &c., on, bib. notice of 733
Manual of pharmacodynamics for physicians, surgeons and students, by M. W. Plagge, M. D., bib. notice of 733
McClellan's principles and practice of surgery, bib. notice of 302
Meigs on females and their diseases, bib. notice of 24
Merit, a handsome tribute to 36
Meek on the physical condition of the aborigines, with an account of their practice of medicine 47
Medical education at Constantinople 77
Meigs' remarks on females and their diseases 121
Meigs' reply to Prof. Simpson's letter on chloroform 148
Medical Practitioner and Student's Library—the Principles and Practice of Midwifery, by D. H. Tucker, M. D. 190
Medical appointments in Philadelphia 306
Medical Association, American 361
" literature in the U. States 405
Medical congress of Italy 456
Medico-legal question 459
Medical chemistry, for the use of students and the profession, by D. Gardner, M. D., bib. notice of 476
Meigs, Dr. J. F., on the diseases of children, bib. notice of 538
Medical science, a dictionary of, by Prof. Dunglison, bib. notice of 639
Medical colleges 646
Medical Examiner, to the patrons of the 761
Medicines, inoculation of 324
Medicine, clinical, Dr. Graves' system of, bib. notice of 638
Medicine, sectional 640
" an inquiry into the degree of certainty in, by Dr. E. Bartlett, bib. notice of 686
Memoranda of anatomy, surgery and physiology, by M. N. Bower, Surgeon, bib. notice of 437
Mercer's contributions to acoustic pathology 444
Meteorology, researches on, by B. Dowler, M. D., bib. notice of 297
Mendenhall's case of sonnambulism 344
Menstruation, at what age does it cease 373
Mitchell on southern versus northern practice 591
Mitchell on yellow fever autopsies 653
Midwifery, the theory and practice of, by F. Churchill, M. D., bib. notice of 300
Military surgery in Paris 388
Miller's case of successful extirpation of the ovarium 545
Minutes of the proceedings of the committee appointed to attend to and alleviate the suffering of the afflicted with malignant fever of 1793, bib. notice of 625
Monneret's history of the cholera, observed in Constantinople during the winter of 1847-8 320
Mortality from phthsis in prisons 329
Müller's principles of physics and meteorology, bib. notice of 244

Naval surgeons 41
Navy, assimilated rank of the civil branch of the 121
Natural history of man, investigation of the theories of the, by F. Van Amringe, bib. notice of 423
Nævus, treatment of 459
Naptha, cure for cholera,—the elixir of Veroniej 518
Neurology and sulphuric ether, by J. B. Flagg, M. D. 15
New publications 122
Neill and Smith's analytical compendium, bib. notice of 683
Nitrate of silver in croup, Dr. Bryan on 342

Obstetric medicine and surgery, prospectus of a bi-monthly record of, edited by Dr. Clay, of Manchester 38

Obituary 123
Obstetrical Remembrancer, or Denman's aphorisms on natural and difficult parturition, augmented by M. Ryan, M. D., bib. notice of 359
Obstetric tables, comprising graphic illustrations, with descriptions and practical remarks, &c., by G. Spratt, M. D., bib. notice of 359
Ocean, greatest ascertained depth of the 652
Ohio medical and surgical journal 646
Opium smoking 132
Opium, mode of detecting small quantities of 710
Ophthalmia, purulent, Dr. Meek on 488
Omo-hyoideus, action of the 711
Orfila's traite de medicine legale, bib. notice of 535
Ossified gall bladder 209
Outlines of human physiology, for primary study and self-instruction, by Dr. G. Valentin, bib. notice of 110
Ovarium, Miller's case of successful extirpation of the 545
Ovarian tumour, spontaneous cure of 650

Patterson's report of cases occurring at the clinic of Jeff. Med. Col. 13
Paine's materia medica and therapeutics, bib. notice of 20
Pathology of synchysis 67
Pathology, acoustic, contributions to, by J. Mercer, M. D. 444
Pathological sequences of acute inflammation of the fibro-mucous structures of the cavity of the tympanum, tympanitis, myringitis 444
Pancoast's case of a large tumour involving the superior maxillary bone 472
Parchappe's anatomico-physiological notes on the structure of the heart of the sturgeon and of the ray 729
Physical condition of the aborigines, with an account of their practice of medicine, by E. G. Meek, M. D. 47
Philadelphia, salubrity of 261
Phthisis, œdema of the lower extremities in, extract of a letter from F. W. Lewis, M. D., to Prof. Mütter 273
Phthisis, another theory on 457
Phthisis and other diseases of Lima, Dr. Smith on 595
Pharmacopœias of Great Britain and the United States, a dispensatory, or commentary on, by R. Christison, M. D., bib. notice of 540
Pharmacodynamics for physicians, surgeons and students, a manual of, by M. W. Plagge, M. D. bib. notice of 733
Pericarditis 397
Poisons in relation to medical jurisprudence and medicine, by A. S. Taylor, F. R. S., bib. notice of 108
Poisons, their symptoms, antidotes, and mode of treatment, a practical treatise on, by O. H. Costill, M. D. bib. notice of 485
Poisoning for burial allowance from a club 561
Poisoning, accidental, with bad calomel, in France 652
Poison from an infusion of oak bark 714
Political distinction of medical men 529
Practice of medicine, Dungl ison on the 33
Prospectus of a bi-monthly record of obstetric medicine and surgery, and of the diseases of women and children, edited by Dr. Clay, of Manchester, 38
Practical physiology for the use of schools and families, by Edward Jarvis, M. D. 197
Principles of physic and meteorology, by Prof. Müller, bib. notice of 244
Principles of medicine, comprising general pathology and therapeutics &c., by C. J. B. Wiliams, bib. notice of 475
Pregnancy, extra-uterine, case of, by S. Peters, M. D. 288
Prolapsus ani, Vincent on the treatment of 325
Prisons, mortality from phthisis in 329
Professional magnanimity 363
Proceedings of the third meeting of the Association of Medical Superintendants of American Institutions for the Insane 439
Proceedings of the annual convention of the Connecticut medical Society, May, 1848, &c., bib. notice of 541
Professors appointed 644
Pruritus ani et vulvæ, treatment of 692
Puerperal fever, in what way it may originate from erysipelas, by R. U. West, M. D. 315
Puerperal fever, illustrations of the contagious nature of, with remarks on its treatment, &c. 506
Pulmonary mucous membrane of amphibia, ciliary motion in, by T. Williams, M. D. 526
Puerperal fever, quinine prophylactic of 709

Purulent opthalmia, by Dr. E. G. Meek 488
Pyrogen, or the electric fluid, Dr. Lake on 371
Pyelites calculosa, Rayer on 601, 659

Quinine, sulphate of, on the alteration of the properties of, by its mixture with coffee, by M. C. F. 327
Quinine prophylactic of puerperal fever 709
Qunia, sulphate of, Dr. Scruggs on the use of 716
Quinine, Dr. Holmes on 763

Rattlesnake, death of Dr. Wainright, from the bite of a 39
Rand on animal charcoal as an antidote 528
Ranking's Half Yearly Abstract of the Medical Sciences, bib. notice of 538
Rayer on pyelitis calculosa 601, 659
Reminiscences of the effects of chlorine largely inhaled, with the favourable result of copious bleeding, by John Redman Coxe, M. D. 211
Researches on the influence of salt added to the food on the development of cattle, by M. Boussingault 324
Researches on the motion of the juices in the animal body, by J. Liebig, bib. notice of 483
Regeneration of the crystaline lens 525
Read's case of super-foetation 527
Retina, formation of images on 590
Report of a curious surgical case 648
Read, Dr., letter from, correcting his case of super-fœtation in a mare 658
Ricord's lecture on blennorrhagia 59
" treatment of secondary syphilis 562
Rima on the radical cure of varices of the inferior extremities 87
Rheumatism 712
Rupture, spontaneous, of the uterus before labour, Dr. Brownbilli's case of 207
Rupture of the fallopian tube 272
Rupture of the heart 767
Rye, ergot of, on the parturient female and her offspring 75
Rye, ergot of, a remedy for excessive dilatation of the pupil from belladonna 708
Ryan on the philosophy of marriage, bib. notice of 689

Salubrity of Philadelphia, 261
Scurvy, Dr. Holmes' remarks on 364
Scruggs on the use of sulphate of quinia 716
Sea sickness, the nature and treatment of, by W. Fisher, M. D. 54
Secondary syphilis, Ricord's treatment of 562
Sectional medicine 640
Seminal diseases, impotency, and other kindred affections, a treatise on the nature of, &c., by Homer Bostwick, M. D., Surgeon, bib. notice of 679
Ship fever, 199
Ship typhus fever in New Orleans 266
Simpson's discovery of a new anæsthetic agent, more efficient than sulphuric ether 70
Simpson on chloroform 148
Simpson's formula for chloroform 147
Singular case of twins 272
Skin diseases, danger of repressing 651
Sloughing of the internal artery, caused by a red hot poker 505
Small pox 37
" " in sheep 65
Smiley's case of hydrophobia 233
Smith's anomalous case of malformation in a child-bearing woman 238
Smith on phthisis and other diseases of Lima 595
Solly on the human brain, bib. notice of 248
Somnambulism, case of, by N. Mendenhall, M. D. 244
Somnolency, remarkable case of 400
Southern versus Northern practice, by B. Rush Mitchell, M. D. 591
Spermatorrhœa, a practical treatise on the causes, symptoms and treatment of, by M. Lallemand, bib. notice of 296
Spasmo-paralysis, on the theory of, in infants and adults, by M. Hall, M. D. 333
Spratt's obstetric tables, bib. notice of 359
State Medical Society 262
" " Convention 306
Stillé's elements of general pathology, bib. notice of 303
Strychnia and veratria, comparative action of 369
Stomach, ulcer of 271
Surgical operations, on the use of ether and chloroform in 18
Surgeons, naval 41

Surgery and midwifery, Cragin on the use of chloroform in 223
Surgery, principles and practice of, by the late Geo. M'Clellan, M. D., bib. notice of 302
Surgery, military, in Paris, 388
Sulphate of quinine, on the alteration of the properties of, by mixture with coffee, 327
Superfœtation, case of, by Dr. A. N. Read 527
Suicide in France, increase of 705
Synchysis, pathology of 67
Syme, Professor, of Edinburgh, 542

Taylor on poisons in relation to medical jurisprudence, bib. notice of 108
Tape-worm, irritable bladder from 651
Tonsils, enlarged, Hamilton's observations on 42
Todd's cyclopedia of anatomy and physiology, bib. notice of 248
Treatment of burns 78
Treatment of fistula in ano 320
Tracts on generation, translated from the German, by C. R. Gilman, M.D. 190
Transactions of the Medical Society of the State of New York, bib. notice of 541
Transactions of the American Medical Association, bib. notice of 740
Tumour, varicose, in the labia during labour 70
Turnbull's remarks on chloroform, 83
Tucker's principles and practice of midwifery 190
Tumour, encephaloid, case of, by G. L. Upshur, M. D. 461
Tumour, ovarian, spontaneous cure of 650
Turnbull's case of cancer in the stomach, with tubercular peritonitis and dropsy of the peritoneum, necroscopy and remarks 724
Typhoid fever, as it prevailed in Germantown, Tennessee, during the fall of 1847, by R. L. Scruggs, M. D. 80
Twins, of extraordinary size, case of, by P. G. Bertolet, M. D. 171
Twins, singular case of 272
Twenty-fifth annual report of the managers of the New York Asylum for lying in women, bib. notice of 437

Ulcer of the stomach 271
Ulcers, callous, of the extremities, Mr. Symes' treatment of 398
United States Marine Hospital 166
University College, London, 362
University of Pennsylvania 542
United States naval surgeons 649
Urine and the blood, Dr. Griffith on, bib. notice of 304
Urine, retention of, cupping-glass in 328
Urine, a simple method of relieving retention of, in diseases of the prostate gland, by Dr. M. C. Bernard 692
Urethra, incision of the 76g
Uterus, cyst in the 69
Uterine hemorrhage, on the prevention of, arising after delivery, from atony of the uterus, by J. Christie, M. D. 512
Upshur's case of encephaloid tumour, 461
Utero-gestation, observations on, by C. Clay, M. D. 569

Varicose tumour of the labia during labour 70
Varices of the inferior extremities, Rima on the radical cure of 87
Valentin's outlines of human physiology, bib. notice of 110
Van Amringe's investigations of the theories of the natural history of man, by Lawrence, Pritchard, and others, founded upon animal analogies, &c., &c., bib. notice of 423
Veratria and strychnia, comparative action of 369
Velpeau on the gravity and treatment of fractures and wounds by fire-arms 557
Venereal diseases, Dr. Bostwick on the nature and treatment of, bib. notice of 679
Vincent on the treatment of prolapsus ani 325
Volition, some of the relations of, to the physiology and pathology of the spinal cord 331
Vomiting caused by relaxation of the abdominal parietes, caused by a bandage 651

Wainright, Dr., death of, from the bite of a ratlesnake 39
Waller on galvanism and electro-magnetism in the treatment of uterine, hemorrhage, and prolapsus uteri 237
Watson's account of a case of the prolapse of the gravid uterus during labour 240
Water commissioners of Boston, report of, on the material best adapted for distribution water pipes, &c. 627
Wallace, Dr., note from 658

Westminster Medical Society 136
Wet nurses and lactation 506
Williams on the ciliary motion in the pulmonary mucous membrane of amphibia 526
Wilson's system of human anatomy, general and special, bib. notice of 640
Wounds, gunshot, Prof. Velpeau on 380
Worthington's annual address to the Chester county Medical Society, bib. notice of 437
Women, Dr. Gooch on the most important diseases peculiar to, bib. notice of 634
Yellow fever in Mexico, Dr. B. R. Mitchell on 285
Yellow fever, Hastings' lectures on, bib. notice of 346
Yellow fever autopsies, by B. Rush Mitchell, M. D. 653
Yearsley on a new mode of treating deafness 576

